# Inaccurate Clinical Thermometers

**Published:** 1905-03

**Authors:** 


### INACCURATE CLINICAL THERMOMETERS.

We learn that since the appearance of the article on Inaccurate Clinical Thermometers, which was published in our issue of October 22d, small dealers from all over the country, from Maine to California and from Minnesota to Florida, have been sending thermometers to the United States Bureau of Standards for testing, that in some cases the proportion of thermometers rejected has been as high as seventy-five per cent., and in many instances the errors observed amounted to seven-tenths of a degree or even more. The results of these investigations are the most convincing evidence that Mr. Mayo did not overestimate the evil of the conditions existing in this department of the surgical instrument trade. The manufacturers seek to evade responsibility by throwing the blame for cheap and necessarily inaccurate thermometers upon physicians and hospital authorities. Unfortunately, the evidence produced indicates a woeful disregard on the part of all concerned of the need for accuracy in these important clinical instruments, and we can not too strongly impress upon our readers that they can not hope to obtain satisfactory instruments at the very low prices which are generally paid for such goods by the hospitals. —*New York Medical Journal and Philadelphia Medical Journal.*

